# Antimicrobial Resistance and Residues from Biofilms in Poultry, Swine, and Cattle Farms: A Scoping Review

**DOI:** 10.3390/ani15182756

**Published:** 2025-09-22

**Authors:** Zehra Irshad, Andrea Laconi, Ronald Vougat Ngom, Roberta Tolosi, Alessandra Piccirillo

**Affiliations:** 1 Department of Comparative Biomedicine and Food Science, University of Padua, 35020 Legnaro, Italy; zehra.irshad@studenti.unipd.it (Z.I.); andrea.laconi@unipd.it (A.L.); roberta.tolosi@unipd.it (R.T.); 2 Department of Animal Production, School of Veterinary Medicine and Sciences, University of Ngaoundéré, Ngaoundéré B.P. 454, Cameroon; romsonbey@yahoo.fr

**Keywords:** antibiotic, antimicrobial residues, antimicrobial resistance, biofilm, cattle, poultry, resistance genes, scoping review, swine

## Abstract

Background: The use of antibiotics in livestock has contributed to the spread of antimicrobial resistance (AMR) and biofilms can play a role in its emergence and dissemination. This review aimed to map the literature on AMR, antimicrobial resistance genes (ARGs), and antibiotic residues (ARs) in biofilms from drinking water distribution systems in poultry, swine, and cattle farms. Methods: The review was conducted according to the PRISMA-ScR extension. Four databases (PubMed, Scopus, Agricola, and Web of Science) were searched. Studies were screened in Rayyan. Results: The search yielded 1242 studies. After screening 732 studies, only 4 met the inclusion criteria. These studies focused on poultry (n = 3) and dairy cattle (n = 1), not on swine. Isolation relied on plating methods. Two studies complemented culturing with 16S rRNA sequencing. No studies applied culture-independent techniques. The number of biofilm-derived isolates across studies ranges from 6 to 102. Three studies performed antimicrobial susceptibility testing, using a wide range of antibiotics (16 to 31). One study analyzed ARGs; none quantified ARs. Conclusions: The limited number of studies and lack of standardized methods hinder the generalizability of the findings, underscoring the need for research to clarify biofilms’ role in AMR dissemination in livestock farms.

## Simple Summary

Scientists are increasingly concerned that the use of antimicrobials in livestock farming, such as poultry, swine, and cattle farming, may contribute to the spread of antimicrobial resistance. One way this resistance may spread is through biofilms, which are bacterial communities that can form inside drinking water pipes on farms. This scoping review looked at past research on antimicrobial resistance and residues in these settings. Out of more than one thousand studies, only four met the inclusion criteria. Three focused on poultry and one on dairy cattle, with none addressing swine. Most of the studies used traditional microbiological methods, and only one study investigated resistance genes. No study measured antimicrobial residues. These findings highlight a critical gap in our knowledge and show that more consistent and thorough research is urgently needed to better understand how biofilms may contribute to antimicrobial resistance on farms, helping us develop safer food systems and protect public health.

## 1. Introduction

The livestock sector plays a crucial role in many countries. Beyond ensuring healthy diets and sustainable food systems, it also contributes to poverty reduction across various population groups [1,2]. Factors such as rising incomes, changing dietary preferences, and population growth have driven increased demand for livestock products, making this sector one of the fastest growing agricultural sub-sectors [3]. However, the intensification of livestock production, characterized by the housing of large numbers of animals in crowded and stressful conditions, facilitates the emergence, transmission, and amplification of diseases, posing significant challenges to their health [4,5]. Livestock diseases are major drivers of antimicrobial use (AMU), with global AMU projected to increase by 8% by 2030 [6]. Currently, 73% of all antimicrobials sold globally are used in food-producing animals [7]. Between 2019 and 2021, annual global AMU was estimated at 76,060 tons of antimicrobial active compounds, with cattle, swine, and poultry accounting for 53.5%, 40.9%, and 5.6%, respectively [8]. AMU can lead to the presence of antimicrobial residues (ARs) in foods of animal origin [1]. Furthermore, the use of antimicrobials in livestock can lead to the emergence of antimicrobial resistance (AMR), which threatens the long-term sustainability of the livestock industry by causing treatment failures in animals [5,6]. Both ARs and AMR can be transferred to humans through the food chain, posing significant public health risks [9,10]. Biofilms are complex, multi-species bacterial communities embedded in an extracellular matrix composed of proteins, polysaccharides, nucleic acids, and water. These structures commonly develop on both abiotic and biotic surfaces [11], providing microbial communities with enhanced resistance to environmental stress, antimicrobials, disinfectants, and host defense mechanisms compared to planktonic bacteria [12,13]. Enhanced AMR within biofilms can be attributed to various mechanisms, such as restricted drug diffusion, internal metabolic processes, and enzyme-mediated effects [14,15]. Biofilms represent a significant challenge in the livestock sector, since they can serve as potential reservoirs for opportunistic and pathogenic bacteria and act as hotspots for horizontal gene transfer, including antimicrobial resistance genes (ARGs) [7,16]. Animal housing facilities and farm equipment, particularly drinking water distribution systems (DWDSs), provide an ideal environment for biofilm formation due to the presence of moisture, nutrients, and organic matter [17,18]. Bacteria within biofilms established in DWDSs can acquire and accumulate ARGs due to high cell densities and close cellular proximity, which facilitate horizontal gene transfer. Over time, resistant bacteria may detach from the biofilms within DWDSs and disseminate into the environment [19]. Antimicrobial drugs (AMDs), which are commonly administrated to large groups of animals through DWDSs, can be captured by biofilms, contributing to treatment failures and promoting the emergence of resistant bacteria [20]. Indeed, Khairullah et al. [21] reported that the antibiotic tolerance of bacteria in biofilms is 100 to 1000 times greater than that of planktonic bacteria. Moreover, biofilms formed by multidrug-resistant (MDR) bacteria represent a serious threat to public health. Therefore, expanding our understanding of biofilms in animal production systems, particularly their impact on antimicrobial treatments and the emergence of AMR, is of the utmost importance. This scoping review aimed to identify and summarize available information on ARs, AMR, and ARGs in biofilms resident in poultry, swine, and cattle farms.

## 2. Materials and Methods

This scoping review was performed following a five-stage framework: (i) identifying the research question, (ii) identifying relevant studies, (iii) study selection, (iv) charting the data, and (v) collating, summarizing, and reporting the results, as described by Levac et al. [22] and reported according to the guidelines stated by the Preferred Reporting Items for Systematic Reviews and Meta-Analyses (PRISMA), extension for Scoping Reviews (PRISMA-ScR) [23]. The details of the PRISMA-ScR checklist are provided as Supplementary Materials S1.

### 2.1. Protocol and Registration

The protocol for this review was developed a priori, archived in the University of Padua Research Archive institutional repository (handle code: https://hdl.handle.net/11577/3519562), and registered on the Systematic Reviews for Animals and Food (SYREAF) website (https://www.syreaf.org/protocol/, accessed on 15 May 2025).

### 2.2. Eligibility Criteria

The inclusion criteria were defined based on the elements of the following PICo elements: observational studies involving poultry, swine, and cattle (Population), focusing on ARs, AMR, and ARGs in biofilms (Interest) within a farm environment (Context). Only primary research studies published in English or French were included, with no restrictions on publication date or geographical location.

### 2.3. Information Sources

Four databases providing a high level of article recalls across biomedical studies [24] were searched. Scopus (Elsevier interface) and Medline (PubMed interface) were accessed via the University of Padua (Padua, Italy), while Agricola (EBSCOhost Research interface) and Web of Sciences (WoS) were accessed via the Baylor University (Waco, TX, USA) library. All WoS databases were used except for those related to proceedings, theses, and social sciences. The search was performed on the 23 April 2024. Additionally, Google Scholar was used to perform a backward search, starting with papers included in the review.

### 2.4. Search Strategy

A multi-strand strategy was applied to ensure high sensitivity [25], combining concepts using the following framework: [Poultry or Cattle or Swine] AND ([Antimicrobial resistance] OR [Antimicrobial resistance genes] OR [Antimicrobial residues]) AND [Biofilm] AND [Farm]. Search terms were adjusted based on the specific database interfaces (Supplementary Materials S2 and S3). All citations retrieved were imported into Zotero software (6.0.36) for deduplication.

### 2.5. Selection Process

Following deduplication, citations were uploaded to Rayyan software (version 1.6.1) for a two-phase screening process, namely screening of titles and abstracts and full-text screening. Six independent reviewers, divided into two groups of three, conducted the screening. Each group reviewed half of the citations, ensuring that every study was screened by three reviewers. Conflicts were initially resolved through discussion among reviewers within the same group; unresolved conflicts were then assessed by a fourth reviewer. To ensure consistency, a calibration exercise on at least 20% of randomly selected papers was conducted by all reviewers at the start of each phase, allowing them to discuss and resolve disagreements before the screening process [26]. For the screening of titles and abstracts, eligibility of studies was assessed using the following criteria:

1. Is the study original research published in English or French? Yes [include], No [exclude], Unclear [include]
2. Does the study involve livestock? Yes [include], No [exclude], Unclear [include]
3. Does the study investigate biofilms from the farm environment? Yes [include], No [exclude], Unclear [include]
4. Does the study focus on ARs, AMR, or ARGs? Yes [include], No [exclude], Unclear [include]

Studies meeting the inclusion criteria at the title and abstract screening passed to the full-text screening. Eligibility of studies was assessed using the following criteria:

1. Is the full text available in English or French? Yes [include], No [exclude]
2. Does the study population include at least one of the following species: poultry, swine, or cattle? Yes [include], No [exclude], Unclear [Exclude]
3. Does the study focus on ARs, AMR, or ARGs in biofilms? Yes [include], No [exclude]
4. Was the study conducted at the farm level? Yes [include], No [exclude]

### 2.6. Data Charting and Items

A Microsoft Excel^®^ 2016 spreadsheet developed by one author and validated by all the others was used for data extraction. All reviewers independently performed data extraction following a procedure similar to the study selection process.

Data collected included general information of studies (i.e., first author; year of publication; duration of the field study; country where the study was conducted; and study design, e.g., cross-sectional, longitudinal study, etc.). Details regarding the population were also collected, including the animal species and production type, such as poultry, swine, and cattle (with distinctions like dairy cattle, calves, heifers, broilers, layers, turkeys, weaners, and finishing pigs). Additionally, the number of farms involved in each study and the type of farm (e.g., conventional, commercial, etc.) were documented. Data concerning biofilm collection were recorded, namely the specific sites where biofilm samples were collected on the farms, the methods used for sampling, the number of samples collected per farm, and the total number of samples collected across the study. Transportation and storage conditions of the samples prior to analysis, as well as the time between sample collection and analysis, were also recorded. Regarding the bacteria of interest (i.e., *E. coli*, *Salmonella* spp., *Campylobacter* spp., *Staphylococcus aureus*, etc.), the methods used for bacterial isolation, antimicrobial susceptibility testing (AST), and DNA/RNA isolation from isolates and/or biofilm samples were also extracted, along with any culture-independent methods used. In terms of ARs and AMR, the collected data included the antimicrobials to which the bacteria were resistant, the proportion of resistant bacteria among the isolates, the ARGs investigated and identified, and the proportion of ARGs detected in biofilm samples. Detection methods used for ARs and the specific residues identified were also considered. For this scoping review, any sample categorized as having intermediate resistance were considered as resistant. The Microsoft Excel^®^ 2016 spreadsheet containing all the extracted variables is available upon request from the corresponding author.

### 2.7. Data Synthesis

The results of the literature search were comprehensively reported, including the total number of citations screened, the duplicates removed, and the number of full-text documents evaluated. A flow diagram was also provided to illustrate the screening process, detailing the reasons for exclusion at the full-text level. All the data collected from the studies that met the inclusion criteria were organized into tables and narratively summarized to provide an overview of the findings.

## 3. Results

### 3.1. Study Selection

**Figure 1:**
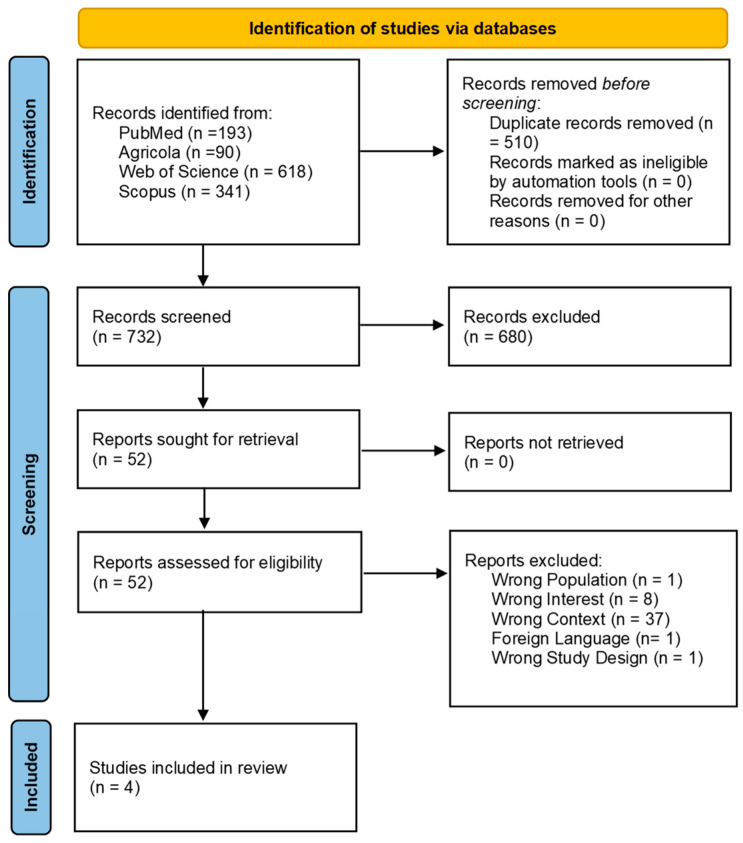
Flow diagram showing the selection process for the scoping review on antimicrobial residues and resistance in biofilms originating from livestock production systems. The diagram outlines each step, from the initial database search to the final inclusion of eligible studies, highlighting reasons for exclusion at various stages.

The search across the four databases resulted in a total of 1242 citations. After duplicates were removed using Zotero, 732 studies were screened based on their title and abstract, and 52 were deemed eligible for the second phase. During the full-text screening, only four studies met the inclusion criteria and were ultimately included in the review (Figure 1). The majority of the studies (n = 37) were excluded because they did not investigate biofilms collected from farm DWDSs, the primary interest of this review.

### 3.2. Study Characteristics

All four studies included in this review were cross-sectional in design and published in English (Table 1).

One study was published in 2021 in Egypt [27], while the other three were published in 2022, from Germany [30], India [29], and Iran [28]. None of the studies reported the type of farming system (e.g., conventional, alternative, etc.). Two of the studies were conducted on broiler farms [28,29], one on layer farms [27], and one on dairy cow farms [30]. Two studies specifically focused on avian pathogenic *E. coli* (APEC) [28,29], collecting samples from broiler farms with mortality linked to colibacillosis. However, only Grakh et al. [29] performed confirmatory analysis (molecular detection of virulence genes) for APEC characterization. While all four studies investigated AMR, none investigated ARs.

**Figure 2:**
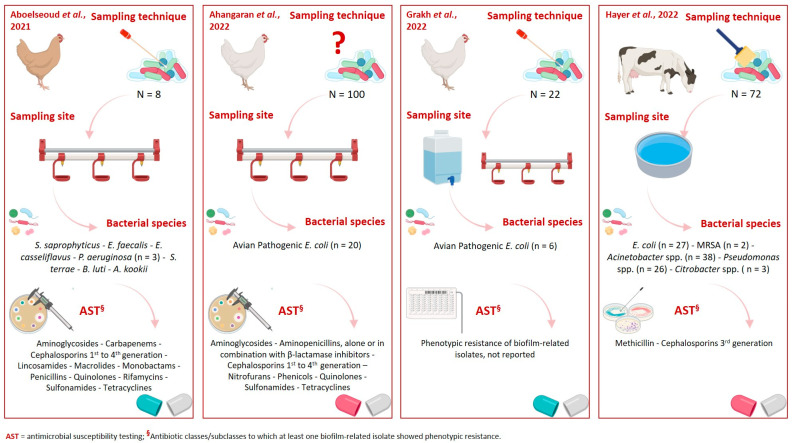
Graphical overview of livestock species, biofilm sampling techniques and sites, bacterial species isolated, and their corresponding phenotypic resistance profiles [27,28,29,30]. Created in https://www.biorender.com/ (accessed on 31 July 2025).

Figure 2 provides a graphical overview of the livestock species, biofilm sampling techniques and sites, bacterial species isolated, and their corresponding phenotypic resistance profiles.

### 3.3. Biofilm Collection, Handling, and Processing

The sampling techniques and locations for biofilm collection varied across the studies (Table 2).

Biofilm samples were collected from DWDSs (i.e., tank, pipelines, troughs, and drinkers), as well as feeders. Three out of the four studies employed swabbing and scraping as methods for biofilm collection. Concerning the shipment and short-term storage conditions of the biofilm samples, two studies reported using ice boxes, and one used an insulated box, with temperatures ranging from 4 °C to 7 °C. One study did not report the sampling technique, transportation, or storage conditions [28]. All biofilm samples were analyzed within 24 h from collection. Only one study [27] provided details on the appearance of biofilm within PVC and iron pipes. While all collected biofilm samples were slimy, the study noted a more prominent, blackish biofilm layer on the inner surface of the PVC pipe compared to the iron pipes. The number of biofilm samples collected in the four studies ranged from 8 to 100, with the number of positive samples varying from 6 to 72 (Table 3).

In the study by Aboelseoud et al. [27], eight biofilm samples were grouped into two pools: pool A, from iron pipelines (*n* = 2), and pool B, from PVC pipelines (*n* = 6). A diverse array of bacterial species was identified across the studies, with *E. coli* being the most frequently studied and the focus of two studies [28,29]. Other species isolated included *Staphylococcus saprophyticus*, *Enterococcus faecalis*, *Enterococcus casseliflavus*, *P. aeruginosa*, *Sphingopyxis terrae*, *Bacillus luti*, and *Acinetobacter kookii* [27]. Additionally, Hayer et al. [30] reported the presence of Methicillin-resistant *S. aureus* (MRSA) and Gram-negative bacteria resistant to third generation cephalosporins (CRB), including *E. coli*, *Acinetobacter* spp., *Pseudomonas* spp., and *Citrobacter* spp. Bacterial isolation was carried out according to standard protocols in two studies: the American Public Health Association guidelines [27] and the DIN EN ISO 6222:1999 and DIN EN ISO 9308-1:2014 standards [30]. Three studies [28,29,30] employed biochemical assays for bacterial identification. Two studies confirmed bacterial species using 16S rRNA sequencing [27,28], while one used end-point PCR targeting the *uspA* gene for *E. coli* confirmation [29]. Notably, Ahangaran et al. [28] and Grakh et al. [29] were the only studies to perform both biochemical and molecular testing. Hayer et al. [30] was the only study that did not include any biomolecular analysis.

### 3.4. Antimicrobial Resistance Profiles of Biofilms

AST was conducted in three out of the four included studies, using two methods: the disk diffusion (DD) test [27,28] and minimum inhibitory concentration (MIC) testing via the Vitek^®^ 2 Compact platform [29] (Table 4).

In the studies by Aboelseoud et al. [27] and Ahangaran et al. [28], the number of isolates tested for AST was 9 and 20, respectively. Grakh et al. [29] did not report the number of biofilm strains tested. In all three studies, the AST results were interpreted according to the Clinical and Laboratory Standards Institute (CLSI) guidelines. Additionally, in Aboelseoud et al. [27], the Multiple Antibiotic Resistance Index (MARI) was assessed. The number of antibiotics tested in these studies varied between 16 [28] and 31 [27], covering 13 different antibiotic classes, including aminoglycosides, beta-lactams, glycopeptides, lincosamides, macrolides, nitrofurans, oxazolidinones, phenicols, polymyxins, potentiated sulphonamides, quinolones, rifamycins, and tetracyclines. Among the antibiotics tested, five (i.e., amikacin, ampicillin, gentamicin, imipenem, and sulfamethoxazole-trimethoprim) were common to all three studies performing AST. The AMR profiles of bacterial strains isolated from biofilms were inconsistently reported in the four studies. For instance, in Grakh et al. [29], it was not possible to extract the resistance profile of the six biofilm strains, since the study reported cumulative data that included isolates from both diseased and healthy birds (*n* = 26) and other environmental sources (*n* = 15). However, since all strains were susceptible to polymyxin B, this information was extrapolated to the biofilm samples. In contrast, Ahangaran et al. [28] provided a detailed resistance profile for *E. coli* isolates (*n* = 20) against each of the antibiotics tested. The highest frequency of resistance was found against tetracyclines, ranging from 25% for doxycycline to 70% tetracycline, followed by sulfamethoxazole-trimethoprim (35%). Full susceptibility was observed against penicillin, imipenem, and gentamicin. Even though Hayer et al. [30] did not perform any AST assays, they assessed the presence of resistant bacterial species, including MRSA and CRB (e.g., *E. coli*, *Acinetobacter* spp., *Pseudomonas* spp., and *Citrobacter* spp.), using selective culture media, such as CHROMagar ESBL and CHROMagar MRSA. In Aboelseoud et al. [27], despite isolating a similar number of bacterial species (*n* = 7), selective culture media were not used, and AST was performed on nine isolates. Among the isolates, *Sphingopyxis terrae* showed the highest number of resistances (i.e., aminoglycosides, beta-lactams, quinolones, potentiated sulphonamides, and tetracyclines). One of the three *P. aeruginosa* strains was susceptible to all the antibiotics tested. Overall, the majority of the isolates (55.5%, *n* = 5) were resistant to at least three antibiotic classes, excluding the three *P. aeruginosa* strains.

### 3.5. Characterization of Resistance- and Biofilm-Related Genes

Molecular methods were employed by Ahangaran et al. [28], which was the only study to investigate the presence of resistant genes using multiplex end-point PCR. The study focused on eight genes (*tetA*, *tetB*, *tetC*, *tetD*, *tetE*, *teG*, *tetK*, *tetL*, *tetM*, *tetO*, and *tetS*), conferring resistance to tetracyclines in isolates (n = 14) showing phenotypic resistance. Among these, only *tetA* (detected in four isolates) and *tetB* (detected in seven isolates) were found, either individually or in combination in at least one isolate. Biofilm-related genes (i.e., *crl*, *csgA*, *fmH*, *luxS*, and *papC*) were investigated by Grakh et al. [29] using end-point PCR. Although specific data on the presence of biofilm-related genes in biofilm isolates could not be extracted, all the investigated genes were detected in at least one sample.

## 4. Discussion

This review examined the available evidence on ARs, AMR, and ARGs in biofilms from poultry, cattle, and swine farms. While an increasing but still limited body of evidence suggests that biofilms may play a role in the persistence and spread of resistant bacteria [13,31,32], our findings highlight a significant research gap regarding biofilms in DWDSs in livestock production due to the limited number of studies retrieved. This gap underscores the need for further research, as understanding the ecology of biofilm-forming bacteria is essential for effective disease prevention in livestock. Only very recently published papers (2021 and 2022) were included in this review. This suggests that the interest in studying biofilms as potential reservoirs or spreaders of AMR has been increasing in recent years [13,31]. Studies were geographically diverse, emphasizing the need for more globally representative studies to understand how geographical, agricultural, and environmental factors may influence biofilm formation and associated AMR [33,34,35]. Studies predominantly focused on poultry [27,28,29], with only one dedicated to dairy cattle [30] and none to swine. This finding suggests the need for future studies that include these species, given their importance as food-producing animals and their role as potential reservoirs of zoonotic and resistant pathogens [36,37]. Additionally, different livestock production systems have specific characteristics that could influence biofilm formation and AMR profiles [38,39]. In particular, swine production represents the projected main livestock species in terms of AMU [40], one of the main drivers of AMR. A notable gap is the lack of studies examining ARs in biofilms, an important area given the role of biofilms in capturing and retaining AMDs, which could contribute to selective pressure and the emergence and persistence of resistant strains on farms and their potential transfer to humans [20,41,42,43,44]. A key consideration in this review is the heterogeneity observed across the biofilm collection and processing methodologies. The sampling techniques varied across studies. This inconsistency complicates comparisons among studies since the sampling methodology has a considerable impact on biofilm research [45]. Since biofilm formation within DWDSs can be influenced by factors such as temperature and water flow [46], the observed variation in sampling (i.e., tank, pipelines, troughs. and drinkers) sites may further hinder the comparability of results across studies. Furthermore, the number of biofilm samples collected in these studies was generally low, with only one study [28] collecting 100 samples. The small and inconsistent sample sizes further limit the generalizability of the findings. Similarly, the significant variation in the protocols used for bacterial isolation and characterization complicates the direct comparison of results across [27,30] studies, suggesting that the adoption of standardized protocols is necessary for accurate and reproducible bacterial recovery from biofilms. Moreover, the methods used for bacterial identification further highlight the diversity of approaches within the field. Biochemical assays were used in three studies [28,29,30], which are often considered time-efficient but may lack the sensitivity and specificity provided by molecular techniques. Three out of four studies [27,28,29] employed molecular methods for bacterial identification, which are highly specific and sensitive methodologies. Without molecular validation, there is a risk of misidentification, which could lead to underestimation or misinterpretation of the bacterial populations present. Despite these limitations, a diverse array of bacterial species was reported, underscoring the complexity and variety of microorganisms associated with biofilms. Among the identified species, *E. coli* emerged as the most frequently studied microorganism. This finding is not surprising given the widespread presence of *E. coli* in both environmental and clinical settings, where it is commonly associated with AMR profiles [47,48]. Specifically, two studies [28,29] focused on APEC in broiler farms, probably due to its importance as a poultry pathogen [49,50]. Furthermore, the occurrence of APEC has been related to its ability to survive under different environmental conditions, which is facilitated by its ability to form biofilms [51,52]. Genes involved in biofilm formation have also been linked to APEC pathogenicity and increased resistance to antimicrobial treatments [53,54]. Other key findings of this review are the considerable variability in the AST methods and antibiotics tested, which may limit the comparability of results across studies [55,56], as well as the scarcity of molecular methods for characterizing the genetic determinants of AMR in livestock-associated biofilms. Several commonly used drugs were included in the ASTs, and among these, some are considered critical for both animal and human health [57], making resistance against them particularly noteworthy. Unfortunately, the small sample size tested in all the studies, as well as the diversity in testing methods, significantly limits the power of the studies and makes it difficult to draw generalizable conclusions about AMR in biofilms across livestock farms. Additionally, Grakh et al. [29] reported only cumulative AMR data, combining findings on strains isolated from both diseased and healthy birds, as well as isolates from various environmental sources. This approach made it impossible to assess the resistance profile. In contrast, Hayer et al. [30] did not provide quantitative resistance data, as no AST assays were performed. The presence of resistant bacterial species (i.e., MRSA and CRB) was assessed using selective culture media. This methodology is recognized as a more sensitive method to detect even small amounts of resistant bacteria, especially in samples like biofilm, where lower bacterial concentrations are frequently present [58]. Regarding molecular characterization of AMR genetic determinants, only one study [28] investigated the presence of *tet* genes in APEC strains phenotypically resistant to tetracyclines. However, the limited number of resistant strains tested (*n* = 14) hindered an accurate estimation of the true prevalence of these genes in poultry farm biofilms. Assessing the prevalence of resistance genes, such as those in the *tet* family, within farm environments is essential for understanding the genetic mechanisms underlying AMR in biofilms and the potential risk of gene transmission to humans, particularly given that these genetic determinants are often located on mobile genetic elements, such as plasmids [59,60]. Finally, only one study [29] investigated biofilm-related genes in *E. coli* and APEC strains. While investigating the presence of these genes in strains isolated from farm biofilms is crucial for understanding how biofilm formation occurs and persists in the farm environment, the retrieved data were insufficient to draw any conclusions. For instance, the number of genes investigated was quite limited (*n* = 5). Furthermore, biofilm formation may be affected not only by the presence of biofilm-related genes but also by their level of expression [61]. This highlights the urgent need for more molecular studies to understand the interplay between AMR and biofilms in farm environments. Notably, none of the retrieved studies employed any advanced characterization methods (e.g., whole-genome sequencing, WGS, etc.) of the isolates, nor did they perform culture-independent analyses (e.g., qPCR, metagenomics, etc.). Future research should prioritize the use of advanced molecular methods, including WGS and transcriptomics, to explore the genetic basis of biofilm formation and resistance mechanisms in farm environments, as well as metagenomics to characterize the complexity and diversity of the biofilm microbiome and resistome. Overall, the variability in sampling and testing methods, sample sizes, and data analysis across the studies highlights the need for more standardized approaches to AMR monitoring in livestock biofilms. Standardized protocols for biofilm collection, processing, and AST would improve the reliability and comparability of results, allowing for a better understanding of AMR dynamics in farm environments. Furthermore, the lack of molecular characterization methods, such as WGS or PCR-based techniques, is a significant gap. Incorporating molecular approaches would provide deeper insights into the genetic basis of AMR and biofilm formation, which are crucial for designing effective prevention strategies. Further research is needed to better understand biofilm formation dynamics, particularly their role in AMR transmission to livestock through drinking water (Table 5).

Overall, this scoping review was subject to certain limitations, primarily due to its focus on peer-reviewed publications, excluding gray literature, preprints, and conference abstracts. Additionally, the quality of the individual studies included may have influenced the findings. For instance, the absence of standardized laboratory methodologies could partly explain the observed variability in reported levels of antimicrobial resistance.

## 5. Conclusions

This scoping review highlights significant gaps in current research on ARs, AMR, and ARGs in biofilms in livestock farming environments. The limited number of studies, their methodological variability, and the absence of standardized protocols for biofilm sampling, processing, and AST hindered the comparability and generalizability of findings. Notably, a critical lack of molecular characterization and no assessment of ARs in biofilm matrices within livestock DWDSs was revealed. As biofilms are increasingly recognized as reservoirs and spreaders of AMR, particularly in DWDSs, future research must prioritize the development of standardized methodologies and incorporate advanced molecular tools to better understand the dynamics of ARs, AMR, and ARGs in livestock-associated biofilms. This is essential for advancing our understanding of AMR in agricultural settings, supporting more informed surveillance efforts, and guiding the development of evidence-based policy recommendations.

## Figures and Tables

**Table 1:** General characteristics of the four studies included in the scoping review.

Reference	Country	Study Design	Animal Species	Number of Farms Visited	Period of Sampling
[27]	Egypt	Cross-sectional	Layer	1	February 2020
[28]	Iran	Cross-sectional	Broiler	20	2019 to 2020
[29]	India	Cross-sectional	Broiler	10	NR
[30]	Germany	Cross-sectional	Dairy Cow	24	March to September 2018

NR = not reported.

**Table 2:** Biofilm samples and sampling techniques reported in the four studies included in the scoping review.

Reference	Sample Location	Sampling Method	Transportation/Storage Conditions	Time to Analysis
[27]	PVC and iron pipelines	Swabbing	Icebox	Within 4 h
[28]	Drinking water distribution systems	NR	NR	NR
[29]	Water tank, water supply pipes, and drinkers	Swabbing	Icebox	Same day
[30]	Troughs	Scraping-off	Insulated box (4 to 7 °C)	Within 24 h

NR = not reported.

**Table 3:** Characteristics of the isolates and methods used for bacterial isolation and identification in the four studies included in the scoping review.

Reference	Total No. of Biofilm Samples	No. of Positive Biofilm Samples	No. of Isolates	Bacterial Species Identified (No. of Isolates)	Methodology Used for Bacterial Isolation and Identification		
					Isolation Method	Identification by Biochemical Assays	Identification by Molecular Assays
[27]	8 *	NA	NA	*Staphylococcus saprophyticus*; *Enterococcus faecalis*; *Enterococcus casseliflavus*; *Pseudomonas aeruginosa* (3); *Sphingopyxis terrae*; *Bacillus luti*; *Acinetobacter kookii*	Heterotrophic bacterial count following the standard protocols of the American Public Health Association	NA	16S rRNA sequencing
[28]	100	20	20	*Escherichia coli*	Direct inoculation of MacConkey and Eosin methylene blue (EMB) agar	IMViC test: indole, methyl red test, Voges–Proskauer test, and citrate utilization test	16S rRNA sequencing
[29]	22	6	6	Avian Pathogenic *Escherichia coli*	Inoculation in brain heart infusion (BHI) broth and MacConkey agar. Inoculation of suspected colonies on EMB	Vitek^®^ 2 Compact	End-point PCR (*uspA* gene)
[30]	72	72 ^	102	*Escherichia coli* (27); Methicillin-resistant *Staphylococcus aureus* (MRSA) (2); Resistant *Escherichia coli* (6); Resistant *Acinetobacter* spp. (38); Resistant *Pseudomonas* spp. (26); Resistant *Citrobacter* spp. (3)	Total bacterial count and enumeration of *Escherichia coli* and coliform bacteria according to DIN EN ISO 6222:1999 and DIN EN ISO 9308-1-2014, respectively. Biofilms diluted at 1:10 in sterile saline solution and filter bags blending. Chromocult coliform agar, CHROMagar ESBL, and CHROMagar MRSA, Columbia sheep blood agar	Oxidase, EnteroPluri or OxiFerm tests	NA

* Biofilm samples were processed in two pools; pool A was collected from iron pipelines (*n* = 2) and pool B from PVC pipelines (*n* = 6). ^ Number of biofilm samples that had positive results for coliform bacteria. Gram-negative bacteria resistant to third generation cephalosporins (CRB) were identified in 49 biofilm samples. NA = not applicable.

**Table 4:** Antimicrobial resistance profiles of bacterial strains isolated from biofilm samples in the four studies included in the scoping review.

		[27]									[28]	[29]
Antibiotic Class/Sub-Class	Active Principle	S. saprophyticus *	E. faecalis *	E. casseliflavus *	P. aeruginosa *	S. terrae *	*B. luti ^#^*	*P. aeruginosa ^#^*	*P. aeruginosa ^#^*	*A. kookii ^#^*	Number (%) of Resistant *E. coli* Strains	*E. coli*
Aminoglycosides	Amikacin	S	-	-	S	R	S	S	S	S	3 (15)	NR
	Gentamycin	S	S	R	S	S	S	S	S	S	0 (0)	NR
	Tobramycin	-	-	-	S	R	-	S	S	S	NA	NR
Aminopenicillins	Ampicillin	-	S	S	-	S	-	-	-	-	5 (25)	NR
	Amoxicillin	NA	NA	NA	NA	NA	NA	NA	NA	NA	6 (30)	NA
Aminopenicillins, in combination with beta-lactamase inhibitors	Amoxicillin-clavulanic acid	-	-	-	-	S	-	-	-	-	2 (10)	NR
Carbapenems	Imipenem	S	-	-	S	S	S	S	S	S	0 (0)	NR
	Ertapenem	-	-	-	-	S	-	-	-	-	NA	NA
	Doripenem	-	-	-	S	R	-	S	S	S	NA	NA
Cephalosporins, 1st- to 4th-generation	Cefalexin	NA	NA	NA	NA	NA	NA	NA	NA	NA	NA	NR
	Cefuroxime	-	-	-	-	R	-	-	-	-	NA	NA
	Ceftriaxone	-	-	-	-	R	-	-	-	R	NA	NA
	Ceftazidime	S	-	-	-	R	R	S	S	R	NA	NA
	Cefpodoxime	R	-	-	-	R	R	-	-	-	NA	NR
	Cefotaxime	-	-	-	-	R	-	-	-	-	NA	NA
	Cefovecin	NA	NA	NA	NA	NA	NA	NA	NA	NA	NA	NR
	Ceftiofur	NA	NA	NA	NA	NA	NA	NA	NA	NA	NA	NR
	Cefepime	S	-	-	S	R	R	S	S	R	NA	NA
	Cefixime	NA	NA	NA	NA	NA	NA	NA	NA	NA	3 (15)	NA
Glycopeptides	Teicoplanin	-	S	S	-	-	-	-	-	-	NA	NA
	Vancomycin	-	S	S	-	-	-	-	-	-	NA	NA
Lincosamides	Clindamycin	R	-	-	-	-	S	-	-	-	NA	NA
Macrolides	Erythromycin	R	R	R	-	-	R	-	-	-	NA	NA
Monobactams	Aztreonam	-	-	-	S	R	-	R	S	-	NA	NA
Nitrofurans	Nitrofurantoin	S	S	S	-	S	S	-	-	-	NA	NR
	Furazolidone	NA	NA	NA	NA	NA	NA	NA	NA	NA	4 (20)	NA
Oxazolidinones	Linezolid	-	S	S	-	-	-	-	-	-	NA	NA
Penicillins	Penicillin	R	S	S	-	-	R	-	-	-	0 (0)	NA
Phenicols	Chloramphenicol	NA	NA	NA	NA	NA	NA	NA	NA	NA	4 (20)	NR
Polymyxins	Polymyxin B	NA	NA	NA	NA	NA	NA	NA	NA	NA	NA	S
Quinolones	Nalidixic Acid	-	-	-	-	R	-	-	-	-	4 (20)	NA
	Ciprofloxacin	S	S	S	S	R	S	S	S	S	6 (30)	NA
	Ofloxacin	S	-	-	S	S	S	R	S	-	NA	NA
	Norfloxacin	R	S	-	S	S	S	S	R	-	NA	NA
	Enrofloxacin	NA	NA	NA	NA	NA	NA	NA	NA	NA	NA	NR
	Marbofloxacin	NA	NA	NA	NA	NA	NA	NA	NA	NA	NA	NR
Rifamycins	Rifampicin	S	R	S	-	-	R	-	-	-	NA	NA
Sulfonamides and dihydrofolate reductase inhibitors combination	Sulfamethoxazole- Trimethoprim	S	-	-	-	R	S	-	-	R	7 (35)	NR
Tetracyclines	Tetracycline	NA	NA	NA	NA	NA	NA	NA	NA	NA	14 (70)	NR
	Doxycycline	R	R	R	-	R	S	-	-	S	5 (25)	NA
	Oxytetracycline	NA	NA	NA	NA	NA	NA	NA	NA	NA	8 (40)	NA
Ureidopenicillins	Piperacillin	-	-	-	S	S	-	S	S	S	NA	NR
Ureidopenicillins, including combinations with beta-lactamase inhibitors	Piperacillin-Tazobactam	-	-	-	S	-	-	-	-	-	NA	NA

“-” = antibiotic not tested against the isolate. * = bacteria from biofilm samples collected from iron pipelines (*n* = 2). ^#^ = bacteria from biofilm samples collected from PVC pipelines. NR = data not retrievable. NA = antibiotic not tested in the study. S: susceptible. R: resistant.

**Table 5:** Strengths, weaknesses, opportunities, and threats (SWOT) related to ARs and AMR in biofilms within DWDSs identified in this scoping review.

Strengths	Weaknesses
Novel and timely topic linking AMR and biofilms in a One Health context	Very limited number of included studies (n = 4), reducing generalizability
Rigorous methodology (PRISMA-ScR compliant and protocol-based)	High heterogeneity in methods, sampling, and bacterial identification
Clear identification of research and knowledge gaps	Minimal synthesis possible due to data inconsistency
	No studies quantified antimicrobial residues (ARs) in biofilms
	Lack of advanced molecular techniques (e.g., WGS, metagenomics) in all studies
**Opportunities**	**Threats**
Development of standardized protocols for biofilm sampling, processing, and AMR testing	Field may remain under-researched without increased funding or policy attention
Focus on underrepresented species like swine and cattle	Continued methodological inconsistency may hamper future meta-analyses
Integration of molecular and culture-independent methods (e.g., WGS, qPCR, metagenomics)	Risk of misinterpretation or overgeneralization due to small evidence base
Interdisciplinary collaboration across microbiology, veterinary, and public health sectors	
Informing global AMR policies and One Health surveillance strategies	

## Data Availability

Data will be available upon reasonable request to the corresponding author.

## Supplementary Materials

The following supporting information can be downloaded at https://www.mdpi.com/article/10.3390/ani15182756/s1: S1: PRISMA-ScR check list; S2: Search strategy used in Agricola; and S3: Search strategy used in Web of science.

## Abbreviations

The following abbreviations are used in this manuscript:

AMDs	Antimicrobial Drugs
AMR	Antimicrobial Resistance
AMU	Antimicrobial Use
APEC	Avian Pathogenic *Escherichia coli*
ARs	Antimicrobial Residues
ARGs	Antimicrobial Resistance Genes
AST	Antimicrobial Susceptibility Testing
BHI	Brain Heart Infusion
CHROMagar ESBL	Chromogenic Agar for Extended-Spectrum Beta-Lactamases
CHROMagar MRSA	Chromogenic Agar for Methicillin-Resistant *Staphylococcus aureus*
CLSI	Clinical and Laboratory Standards Institute
CRB	Cephalosporin-Resistant Bacteria
DD	Disk Diffusion
DIN	Deutsches Institut für Normung (German Institute for Standardization)
DWDSs	Drinking Water Distribution Systems
EMB	Eosin Methylene Blue
IMViC	Indole, Methyl Red, Voges-Proskauer, Citrate
ISO	International Organization for Standardization
MARI	Multiple Antibiotic Resistance Index
MIC	Minimum Inhibitory Concentration
MRSA	Methicillin-Resistant *Staphylococcus aureus*
PICo	Population, Interest, Context
PRISMA-ScR	Preferred Reporting Items for Systematic Reviews and Meta-Analyses Extension for Scoping Reviews
PVC	Polyvinyl Chloride
SWOT	Strengths, Weaknesses, Opportunities, and Threats
SYREAF	Systematic Reviews for Animals and Food
WHO	World Health Organization
WoS	Web of Science
WGS	Whole Genome Sequencing
