# Supplementary material for: Antimicrobial Resistance and Residues from Biofilms in Poultry, Swine, and Cattle Farms: A Scoping Review
Source: Animals (Basel). 2025 Sep 22;15(18):2756. doi: 10.3390/ani15182756 (PMC12466552; doi:10.3390/ani15182756)
Supplement: Supplementary file 1 [file animals-15-02756-s001.zip › Supplementary material S2.pdf]

EBSCOhost

Baylor University Libraries

Search History/Alerts

Print Search History | Retrieve Searches | Retrieve Alerts | Save Searches / Alerts

Select / deselect all | Search with AND | Search with OR | Delete Searches

Refresh Search Results

Search ID#	Search Terms	Search Options	Actions
S11	S1 AND S6 AND S7 AND S10	**Expanders** - Apply equivalent subjects **Search modes** - Boolean/ Phrase	View Results (90) View Details Edit
S10	S4 OR S8 OR S9	**Expanders** - Apply equivalent subjects **Search modes** - Boolean/ Phrase	View Results (109,421) View Details Edit
S9	S2 AND S5	**Expanders** - Apply equivalent subjects **Search modes** - Boolean/ Phrase	View Results (18,491) View Details Edit
S8	S2 AND S3	**Expanders** - Apply equivalent subjects **Search modes** - Boolean/ Phrase	View Results (77,330) View Details Edit

☐	S7	TI (farm* or “farm-level”) OR AB (farm* or “farm-level”)	**Expanders** - Apply equivalent subjects<br>**Search modes** - Boolean/ Phrase	View Results (234,711)<br>View Details<br>Edit
☐	S6	TI biofilm* OR AB biofilm*	**Expanders** - Apply equivalent subjects<br>**Search modes** - Boolean/ Phrase	View Results (26,025)<br>View Details<br>Edit
☐	S5	TI residue* OR AB residue*	**Expanders** - Apply equivalent subjects<br>**Search modes** - Boolean/ Phrase	View Results (128,414)<br>View Details<br>Edit
☐	S4	TI (("resistance gene*" OR ARG OR "AMR gene*" OR "resistance determinant*" OR "mobile genetic element*" OR MGE) OR AB (("resistance gene*" OR ARG OR "AMR gene*" OR "resistance determinant*" OR "mobile genetic element*" OR MGE)	**Expanders** - Apply equivalent subjects<br>**Search modes** - Boolean/ Phrase	View Results (25,126)<br>View Details<br>Edit
☐	S3	TI (resistance OR resistant OR sensibility OR susceptibility) OR AB (resistance OR resistant OR sensibility OR susceptibility)	**Expanders** - Apply equivalent subjects<br>**Search modes** - Boolean/ Phrase	View Results (352,460)<br>View Details<br>Edit
☐	S2	TI (multidrug OR MDR OR "multi-drug" OR drug OR antibiotic* OR antimicrobial* OR "anti-microbial*" OR microbial* OR antibacterial* OR "anti-bacterial*" OR	**Expanders** - Apply equivalent	View Results (505,017)<br>View Details

		bacteria$) OR AB (multidrug OR MDR OR "multi-drug" OR drug OR antibiotic* OR antimicrobial* OR "anti-microbial*" OR microbial* OR antibacterial* OR "anti-bacterial*" OR bacteria$)	subjects **Search modes** - Boolean/ Phrase	Edit
☐	S1	TI (pig* OR swine* OR weaner OR fattener OR sow OR piglet* OR boar OR boars OR chick* OR poultry* OR broiler* OR layer* OR turkey* OR duck* OR geese OR goose OR fowl* OR avian* OR bird* OR hen OR hens OR flock* OR cattle OR beef OR cow* OR calf OR calves OR heifer* OR bull* OR bovine OR dairy OR "food-producing animal*" OR "food producing animal*" OR "food animal*" OR "animal husbandry" OR "animal farming" OR "domestic animal*" OR livestock) OR AB (pig* OR swine* OR weaner OR fattener OR sow ...	**Expanders** - Apply equivalent subjects **Search modes** - Boolean/ Phrase	View Results (951,464) View Details Edit

1. Characteristics, Whole-Genome Sequencing and Pathogenicity Analysis of Escherichia coli from a White Feather **Broiler Farm**

By: Wu, Shaopeng ; Cui, Lulu ; Han, Yu ; Lin, Fang ; Huang, Jiaqi ; Song, Mengze ; Lan, Zouran ; Sun, Shuhong . In *Microorganisms.* 2023 11(12) Language: English. DOI: 10.3390/microorganisms11122939

Subjects: Escherichia coli; **antibiotic resistance**; **antibiotics**; **biofilm**; **birds**; death; enterotoxins; **farms**; **food** chain; **food** safety; human health; intraperitoneal injection; monitoring; mortality; multiple **drug resistance**; phylogeny; **poultry**; **poultry** production; public health; virulence; China

BU InfoLinks

2. Diverse Genotypes of Cronobacter spp. Associated with **Dairy Farm** Systems in Jiangsu and Shandong Provinces in China

By: Liu, Hui ; Ji, Xing ; Sun, Haichang ; Billington, Craig ; Hou, Xiang ; Soleimani-Delfan, Abbas ; Wang, Ran ; Wang, Heye ; Zhang, Lili . In *Foods.* 2024 13(6) Language: English. DOI: 10.3390/foods13060871

Subjects: Cronobacter sakazakii; **antibiotic resistance**; **biofilm**; cephalothin; cross contamination; **dairy farming**; dried milk; endotoxins; environmental fate; feces; **food** pathogens; fosfomycin; genomics; genotype; immune evasion; infant formulas; loci; milk; monitoring; phylogeny; plasmids; raw milk; secretion; serotypes; silage; stress tolerance; virulence; China

BU InfoLinks

3. Occurrence of **multidrug resistance** associated with extended-spectrum β-lactamase and the **biofilm** forming ability of Escherichia coli in environmental **swine husbandry**

By: Mitsuwan, Watcharapong; Intongead, Sutsiree; Saengsawang, Phirabhat; Romyasamit, Chonticha; Narinthorn, Ruethai; Nissapatorn, Veeranoot; Pereira, Maria de Lourdes; Paul, Alok K.; Wongtawan, Tuempong; Boripun, Ratchadaporn. In *Comparative Immunology, Microbiology and Infectious Diseases.* 2023 103 Language: English. DOI: 10.1016/j.cimid.2023.102093

Subjects: Escherichia coli; adhesion; ampicillin; **antibiotic resistance**; beta-lactamase; beta-lactamase **bacteria**; **biofilm**; cephalosporins; feces; **genes**; immunology; multiple **drug resistance**; **swine**; tetracycline; wastewater; Thailand; **Biofilms**; Environment; Extended-spectrum beta-lactamase; **Swine farms**

BU InfoLinks

4. **Bovine** Lactoferrin and **Hen** Ovotransferrin Affect Virulence Factors of Acute Hepatopancreatic Necrosis Disease (AHPND)-Inducing Vibrio parahaemolyticus Strains

By: Vandeputte, Marieke ; Verhaeghe, Margaux ; Willocx, Lukas ; Bossier, Peter ; Vanrompay, Daisy . In *Microorganisms.* 2023 11(12) Language: English. DOI: 10.3390/microorganisms11122912

Subjects: Vibrio parahaemolyticus; **antibiotic resistance**; **bacterial** growth; **biofilm**; carboxylic ester hydrolases; disease control; growth retardation; **hens**; hydrophobicity; industry; lactoferrin; necrotizing hepatopancreatitis; pollution; shrimp; virulence

BU InfoLinks

5. Isolation of Pathogenic **Bacteria** from **Dairy Cow** Mastitis and Correlation of **Biofilm** Formation and **Drug Resistance** of Klebsiella pneumoniae in Jiangsu, China

By: Cao, Wendi ; Xu, Yi ; Huang, Yicai ; Xu, Tianle . In *Agriculture.* 2023 13(10) Language: English. DOI: 10.3390/agriculture13101984

Subjects: Klebsiella pneumoniae; agriculture; amoxicillin; beta-lactamase **bacteria**; **biofilm**; **dairy cows**; disease prevention; **drug resistance**; epidemiology; **genes**; gentamicin; gentian violet; mastitis; milk; phenotype; piperacillin; tetracycline; China

BU InfoLinks

6. High incidence of multiple intI1 genomic **gene** cassettes in Aeromonas strains

By: Bo Ling; Feng, Wei; Yang, Nuo; Fan, Lixia; Guo, Guiying; Li, Xuesong; Zeng, Jifeng; Zheng, Jiping. In *Aquaculture.* 2024 579 Language: English. DOI: 10.1016/j.aquaculture.2023.740171

Subjects: Aeromonas; Lithobates catesbeianus; **antibiotic resistance**; **antimicrobial** peptides; aquaculture; **biofilm**; **bullfrogs**; fluoroquinolones; **genes**; genomics; multiple **drug resistance**; rifampicin; spectinomycin; streptomycin; trimethoprim; Aeromonas spp; Rana catesbeiana; Class 1 integron; **Gene** cassette

BU InfoLinks

7. **The Efficacy of Bacteriocins Against Biofilm-Producing Bacteria Causing Bovine Clinical Mastitis in Dairy Farms: A New Strategy**

By: Raheel, Ismail; Mohammed, Asmaa N.; Mohamed, Asmaa Abdrabo. In *Curr Microbiol.* 2023 80(7 p.229-229):229-229. Language: English. DOI: 10.1007/s00284-023-03324-x

Subjects: Bacillus subtilis; Streptococcus; agar; bacteriocins; **biofilm**; **bovine** mastitis; **cattle**; coagulase negative staphylococci; etiology; milk

Linked Full Text BU InfoLinks **BU InfoLinks**

8. **Antimicrobial Resistance and Biofilm Formation of Escherichia coli Isolated from Pig Farms and Surroundings in Bulgaria**

By: Kaleva, Mila D.; Ilieva, Yana ; Zaharieva, Maya Margaritova; Dimitrova, Lıudmila,; Kim, Tanya Chan; Tsvetkova, Iva ; Georgiev, Yordan ; Orozova, Petya ; Nedev, Krasimir ; Najdenski, Hristo . In *Microorganisms.* 2023 11(8) Language: English. DOI: 10.3390/microorganisms11081909

Subjects: Escherichia coli; Shiga toxin; aminoglycosides; **animal husbandry**; **antibiotic resistance**; **antibiotics**; **biofilm**; desorption; disease prevention; disk diffusion **antimicrobial** test; enterotoxins; feces; growth promotion; issues and policy; macrolides; mass spectrometry; medicine; plasmids; polymerase chain reaction; quinolones; saprophytes; virulence; wastewater; Bulgaria

BU InfoLinks **BU InfoLinks**

9. **Prevalence, Serotypes, Antimicrobial Resistance and Biofilm-Forming Ability of Listeria monocytogenes Isolated from Bulk-Tank Bovine Milk in Northern Greece**

By: Angelidis, Apostolos S.; Grammenou, Afroditi S.; Kotzamanidis, Charalampos ; Giadinis, Nektarios D.; Zdragas, Antonios G.; Sergelidis, Daniel . In *Pathogens.* 2023 12(6) Language: English. DOI: 10.3390/pathogens12060837

Subjects: Listeria monocytogenes; **antibiotic resistance**; bulk milk; **cattle**; clindamycin; **farms**; milk; multiple **drug resistance**; pathogens; penicillins; public health; serotypes; virulence; Greece

BU InfoLinks **BU InfoLinks**

10. Characterization and pathological analysis of Flavobacterium tructae recovered from **farmed** rainbow trout, Oncorhynchus mykiss (Walbaum, 1792), in the Indian Himalayan Region

By: Mallik, Sumanta Kumar; Singh, Shivam; Shahi, Neetu; Pathak, Richa; Krishna, Kala,; Das, Partha; Singh, Bhupendra; Giri, Abhay Kumar; Chandra, Suresh; Sarma, D.; Pandey, Pramod Kumar. In *Aquacult Int.* 2023 31(4 p.2399-2420):2399-2420. Language: English. DOI: 10.1007/s10499-023-01089-5

Subjects: Flavobacterium; Oncorhynchus mykiss; alanine transaminase; aminoglycosides; **antibiotic resistance**; aspartate transaminase; **bacteria**; **biofilm**; blood serum; disease progression; erythromycin; fish culture; florfenicol; folic acid; hemolysis; histopathology; kidneys; liver; mortality; muscles; opportunistic pathogens; oxytetracycline; phylogeny; risk; spleen; tetracycline; therapeutics; trout; virulence; Himalayan region

Linked Full Text **BU InfoLinks**

11. Host receptor identification of a polyvalent lytic phage GSP044, and preliminary assessment of its efficacy in the clearance of Salmonella

By: Gao, Dongyang; Ji, Hongyue; Li, Xin; Ke, Xiquan; Li, Xiangmin; Chen, Pin; Qian, Ping. In *Microbiological Research.* 2023 273 Language: English. DOI: 10.1016/j.micres.2023.127412

Subjects: DNA; Escherichia coli; Salmonella; **antibiotics**; **bacteria**; bacteriophages; **biofilm**; **chickens**; **farms**; gastrointestinal diseases; genome; host range; intestines; latent period; mice; models; nucleotide sequences; outer membrane proteins; pH; phylogeny; research; serotypes; sewage; **swine**; terminase; Phage; Phage receptor; Polyvalent

BU InfoLinks

12. Association between **antimicrobial resistance** and **biofilm** forming ability of Salmonella enterica serotypes from commercial **broiler farms** in Brazil

By: Voss-Rech, D.; Ziech, R. E.; Vaz, C. S. L.; Coldebella, A.; Kuchiishi, S. S.; Balzan, C.; Matter, L.; Vargas, Á. C.; Botton, S. A. In *British* ***Poultry*** *Science.* 2023 64(2 p.224-230):224-230. Language: English. DOI: 10.1080/00071668.2022.2136511

Subjects: Salmonella enterica; ampicillin; **antibiotic resistance**; **biofilm**; **farms**; multiple **drug resistance**; nalidixic acid; **poultry** science; serotypes; tetracycline; Brazil; Salmonella; **multidrug resistance**; **broilers**; isolation; serotyping

BU InfoLinks

13. Characterization and **antimicrobial susceptibility** of **biofilm-producing Avian** Pathogenic Escherichia coli from **broiler chickens** and their environment in India

By: Grakh, Kushal; Mittal, Dinesh; Prakash, Anand; Jindal, Naresh. In *Veterinary research communications.* 2022 46(2 p.537-548):537-548. Language: English. DOI: 10.1007/s11259-021-09881-5

Subjects: Escherichia coli; agar; **anti**-infective agents; **antibiotic resistance**; **biofilm**; cloaca; **gene** amplification; heart; indigenous species; lactose; liver; multiple **drug resistance**; pathogenesis; **poultry** industry; selective media; veterinary medicine; virulence; India

Linked Full Text BU InfoLinks

14. Isolation and evaluation of the efficacy of bacteriophages against **multidrug-resistant** (**MDR**), methicillin-**resistant** (MRSA) and **biofilm-producing** strains of Staphylococcus aureus recovered from **bovine** mastitis

By: Mohammadian, Fatemeh; Rahmani, Hamideh Kalateh; Bidarian, Behnam; Khoramian, Babak. In *BMC Vet Res.* 2022 18(1 p.406-406):406-406. Language: English. DOI: 10.1186/s12917-022-03501-3

Subjects: Podoviridae; **antibiotics**; bacteriophages; biological control; **bovine** mastitis; fermented milk; latent period; methicillin-**resistant** Staphylococcus aureus; milk; multiple **drug resistance**; sewage

HTML Full Text PDF Full Text BU InfoLinks

15. **Anti**-virulence compounds against Staphylococcus aureus associated with **bovine** mastitis: A new therapeutic option?

By: Sabino, Yasmin Neves Vieira; Cotter, Paul David; Mantovani, Hilario C. In *Microbiological Research.* 2023 271 Language: English. DOI: 10.1016/j.micres.2023.127345

Subjects: Staphylococcus aureus; **antibiotic resistance**; **biofilm**; **bovine** mastitis; **cattle**; cell viability; **dairy** industry; mammary glands; microorganisms; pathogens; research; therapeutics; traditional medicine; virulence; Quorum sensing; Screening; Toxins; Virulence factors

BU InfoLinks

16. Bacillus cereus in the Artisanal Cheese Production Chain in Southwestern Mexico

By: Cruz-Facundo, Itzel-Maralhi ; Toribio-Jiménez, Jeiry ; Castro-Alarcón, Natividad ; Leyva-Vázquez, Marco-Antonio ; Rodríguez-Ruíz, Hugo-Alberto ; Pérez-Olais, José-Humberto ; Adame-Gómez, Roberto ; Rodríguez-Bataz, Elvia ; Reyes-Roldán, Joel ; Muñoz-Barrios, Salvador ; Ramírez-Peralta, Arturo . In *Microorganisms.* 2023 11(5) Language: English. DOI: 10.3390/microorganisms11051290

Subjects: Bacillus cereus; agar; air; **antibiotic resistance**; artisan cheese; beta-lactams; **biofilm**; cheesemaking; **dairy farming**; egg yolk; **farms**; folic acid; **genes**; genotyping; mannitol; milk; minimum inhibitory concentration; phylogeny; polymyxins; traditional technology; Mexico

BU InfoLinks

17. Genotypic and phenotypic diversity of Prototheca spp. recovered from **bovine** mastitis in terms of **antimicrobial resistance** and **biofilm** formation ability

By: Tashakkori, Niloufar; Rahmani, Hamideh Kalateh; Khoramian, Babak. In *BMC Vet Res.* 2022 18(1 p.452-452):452-452. Language: English. DOI: 10.1186/s12917-022-03546-4

Subjects: Prototheca; amphotericin B; **antibiotic resistance**; **biofilm**; **bovine** mastitis; **cattle**; colistin; etiological agents; **genetic** variation; gentamicin; pathogenicity; phenotypic variation; random amplified polymorphic DNA technique; therapeutics

HTML Full Text PDF Full Text BU InfoLinks

18. **Antimicrobial resistance**, **biofilm** production and invasion of mammary epithelial cells by Enterococcus faecalis and Enterococcus mundtii strains isolated from **bovine** subclinical mastitis in Brazil

By: Rodrigues, D.S.; Lannes-Costa, P.S.; Santos, G.S.; Ribeiro, R.L.; Langoni, H.; Teixeira, L.M.; Nagao, P.E. In *Letters in applied microbiology.* 2022 75(2 p.184-194): 184-194. Language: English. DOI: 10.1111/lam.13718

Subjects: Enterococcus faecalis; Enterococcus mundtii; actin; **antibiotic resistance**; **biofilm**; **cattle**; cytoskeleton; **dairy** industry; epithelium; erythromycin; **food** chain; fosfomycin; linezolid; mammary glands; matrix-assisted laser desorption-ionization mass spectrometry; microbiology; milk; multiple **drug resistance**; norfloxacin; public health; pulsed-field gel electrophoresis; tetracycline; Brazil

Linked Full Text **BU InfoLinks**

19. The prevalence and characteristics of extended-spectrum β-lactamase Escherichia coli in raw milk and **dairy farms** in Northern Xinjiang, China

By: Huang, Shudi; Tian, Peng; Kou, Xiaomeng; An, Ning; Wu, Yushuang; Dong, Juan; Cai, Huixue; Li, Baokun; Xue, Yawen; Liu, Yuezhang; Ji, Hua. In *International journal of **food** microbiology.* 2022 381 Language: English. DOI: 10.1016/j.ijfoodmicro.2022.109908

Subjects: **biofilm**; **drug resistance**; extraintestinal pathogenic Escherichia coli; **food** microbiology; **genes**; **genetic** variation; phylogeny; pulsed-field gel electrophoresis; raw milk; virulence; China; Extended-spectrum β-lactamase; **Antibiotic resistance**; Virulence-associated **genes**; **Biofilm**; Phylogenic group; Pulsed field gel electrophoresis

BU InfoLinks

20. Prevalence, **Antimicrobial Resistance**, and Characterization of Staphylococcus aureus Isolated from Subclinical **Bovine** Mastitis in East Coast Malaysia

By: Saeed, Shamsaldeen Ibrahim; Mat Yazid, Khairun Anisa; Hashimy, Hidayatul Athirah; Dzulkifli, Siti Khadijah; Nordin, Fatihah ; Nik Him, Nik Azmi; Omar, Mohd Fikry Fahmi bin; Aklilu, Erkihun ; Mohamad, Maizan ; Zalati, Che Wan Salma; Kamaruzzaman, Nor Fadhilah. In ***Animals***. 2022 12(13) Language: English. DOI: 10.3390/ani12131680

Subjects: ampicillin; **antibiotic resistance**; **biofilm**; **bovine** mastitis; coasts; **drug** therapy; epithelium; erythromycin; gentamicin; milk; oxacillin; pathogens; somatic cells; tetracycline; Malaysia

HTML Full Text PDF Full Text BU InfoLinks

21. **Farm** Biosecurity Measures and Interventions with an Impact on **Bacterial Biofilms**

By: Butucel, Eugenia ; Balta, Igori ; McCleery, David ; Morariu, Florica ; Pet, Ioan ; Popescu, Cosmin Alin; Stef, Lavinia ; Corcionivoschi, Nicolae . In *Agriculture (Basel).* 2022 12(8) Language: English. DOI: 10.3390/agriculture12081251

Subjects: agriculture; **antibiotic resistance**; biochemical pathways; biocides; **biofilm**; biosecurity; coumarins; **farm** management; **farms**; flavonoids; **livestock**; oleic acid; oregano

BU InfoLinks

22. Prevalence of Escherichia coli strains in horticultural **farms** from Argentina: **antibiotic resistance**, **biofilm** formation, and phylogenetic affiliation

By: Pellegrini, María Celeste; Okada, Elena; González Pasayo, Ramón Alejandro; Ponce, Alejandra Graciela. In *Environmental science and pollution research.* 2022 29(16 p.23225-23236):23225-23236. Language: English. DOI: 10.1007/s11356-021-17523-1

Subjects: Escherichia coli; amoxicillin; **antibiotic resistance**; **biofilm**; environmental assessment; fresh produce; groundwater; horticulture; minimum inhibitory concentration; pathogens; phenotype; phylogeny; pollution; polymerase chain reaction; **poultry** manure; research; soil; vegetables; Argentina

HTML Full Text PDF Full Text BU InfoLinks

23. Salmonella enterica Serovar Typhimurium and Enteritidis Isolated from Raw Shrimp in Bangladesh: An Investigation Based on Molecular Characteristics, Survival, Virulence, **Antibiotic Resistance**, and **Biofilm** Formation Attributes

By: Das, Prosanto Kumar; Mandal, Ananta; Rahman, Md. Mizanur; Sarkar, Shovon Lal; Jahid, Iqbal Kabir; Hossain, Md. Anwar; Alam, A. S. M. Rubayet Ul; Roy, Pravas Chandra. In *Journal of **food** quality.* 2022 2022 Language: English. DOI: 10.1155/2022/3420364

Subjects: Salmonella Typhimurium; **antibiotic resistance**; bile; bile **resistance**; **biofilm**; digestive tract; **food** quality; habituation; humans; income; pathogens; **poultry**; serotypes; shrimp; shrimp fisheries; small intestine; stomach; virulence; Bangladesh

HTML Full Text **PDF Full Text** **BU InfoLinks**

24. Efficacy and safety of phage therapy against Salmonella enterica serovars Typhimurium and Enteritidis estimated by using a battery of in vitro tests and the Galleria mellonella **animal** model

By: Kosznik-Kwaśnicka, Katarzyna; Stasiłojć, Małgorzata; Grabowski, Łukasz; Zdrojewska, Karolina; Węgrzyn, Grzegorz,; Węgrzyn, Alicja. In *Microbiological research.* 2022 261 Language: English. DOI: 10.1016/j.micres.2022.127052

Subjects: Galleria mellonella; Salmonella enterica; **animal** models; **antibiotic resistance**; **bacteria**; bacteriophages; **biofilm**; cell culture; **chickens**; fibroblasts; **food** pathogens; phage therapy; research; serotypes; viability assays; Phage therapy; Salmonella infection; **Poultry**; **Biofilm** reduction; Phage cocktail

BU InfoLinks

25. **Antimicrobial** and antibiofilm potentials of cinnamon oil and silver nanoparticles against Streptococcus agalactiae isolated from **bovine** mastitis: new avenues for countering **resistance**

By: Abd El-Aziz, Norhan K.; Ammar, Ahmed M.; El-Naenaeey, El-sayed Y. M.; El Damaty, Hend M.; Elazazy, Asmaa A.; Hefny, Ahmed A.; Shaker, Asmaa; Eldesoukey, Ibrahim E. In *BMC veterinary research.* 2021 17(1 p.136-136):136-136. Language: English. DOI: 10.1186/s12917-021-02842-9

Subjects: Streptococcus agalactiae; **anti**-infective agents; **antibiotic resistance**; **biofilm**; biosynthesis; **bovine** mastitis; **cattle** industry; cinnamon oil; fimbriae; milk; multiple **drug resistance**; nanosilver; pathogens; plankton; reverse transcriptase polymerase chain reaction; therapeutics; veterinary medicine; virulence; Egypt

HTML Full Text **PDF Full Text** **BU InfoLinks**

26. Novel Streptococcus uberis sequence types causing **bovine** subclinical mastitis in Hainan, China

By: Zeng, Jifeng; Wang, Yu; Fan, Lixia; Yang, Nuo; Pan, Jiwen; Han, Yu; Wang, Xin; Li, Qian; Guo, Guiying; Zheng, Jiping; Zeng, Weixin. In *Journal of applied microbiology.* 2022 132(3 p.1666-1674):1666-1674. Language: English. DOI: 10.1111/jam.15235

Subjects: Streptococcus uberis; **anti**-infective agents; **antibiotic resistance**; **biofilm**; **cattle**; microbiology; milk; molecular epidemiology; multilocus sequence typing; multiple **drug resistance**; virulence; China

Linked Full Text **BU InfoLinks**

27. Occurrence and characterization of coagulase positive and negative Staphylococci isolated from Japanese quails and **broiler chickens** at Qena Governorate, Egypt

By: Younis, Waleed; Sabra, Mahmoud; Sayed, Haitham Helmy. In *SVU-International Journal of Veterinary Sciences.* 2021 4(4 p.1-15):1-15. Language: English. DOI: 10.21608/SVU.2021.92987.1146

Subjects: Coturnix japonica; Staphylococcus aureus; agar; amoxicillin; **antibiotic resistance**; **biofilm**; **broiler chickens**; cefazolin; cefotaxime; chloramphenicol; ciprofloxacin; clavulanic acid; clindamycin; erythromycin; gentamicin; humans; methicillin; multiple **drug resistance**; oxacillin; oxytetracycline; phenotype; vancomycin; Egypt; characterization; coagulase positive; coagulase negative; staphylococci

BU InfoLinks

28. Novel Polyherbal Nanocolloids to Control **Bovine** Mastitis

By: Ranjani, S.; Priya, P. Shruthy; Veerasami, Maroudam; Hemalatha, S. In *Applied biochemistry and biotechnology.* 2022 194(1 p.246-265):246-265. Language: English. DOI: 10.1007/s12010-021-03748-w

Subjects: Acinetobacter baumannii; Acinetobacter junii; Cinnamomum verum; Cymbopogon citratus; Klebsiella pneumoniae; Phyllanthus emblica; Pseudomonas stutzeri; Syzygium aromaticum; Terminalia chebula; **antibacterial** properties; **antibiotic resistance**; **biofilm**; biotechnology; **bovine** mastitis; colloids; cost effectiveness; **dairy cattle**; medicine; milk; minimum inhibitory concentration; multiple **drug resistance**; virulence

Linked Full Text BU InfoLinks

29. Comparative occurrence of ESBL/AmpC beta-lactamase-**producing** Escherichia coli and Salmonella in contract **farm** and backyard **broilers**

By: Chowdhury, M.; Bardhan, R.; Pal, S.; Banerjee, Aparna; Batabyal, K.; Joardar, S.N.; Mandal, G.P.; Bandyopadhyay, S.; Dutta, T.K.; Sar, T.K.; Samanta, I. In *Letters in applied microbiology.* 2022 74(1 p.53-62):53-62. Language: English. DOI: 10.1111/lam.13581

Subjects: Escherichia coli; Salmonella; ampicillin; **antibiotic resistance**; beta-lactamase; **biofilm**; chloramphenicol; cloaca; colistin; databases; doxycycline; **farms**; gentamicin; microbiology; **poultry** meat; tetracycline; India

Linked Full Text BU InfoLinks

30. **Antibiotic Resistance** of Staphylococci from Bulk-Tank Milk of Sheep **Flocks**: Prevalence, Patterns, Association with **Biofilm** Formation, Effects on Milk Quality, and Risk Factors

By: Lianou, Daphne T.; Petinaki, Efthymia ; Cripps, Peter J.; Gougoulis, Dimitris A.; Michael, Charalambia K.; Tsilipounidaki, Katerina ; Skoulakis, Anargyros ; Katsafadou, Angeliki I.; Vasileiou, Natalia G. C.; Giannoulis, Themis ; Voidarou, Chrysoula ; Mavrogianni, Vasia S.; Caroprese, Mariangela ; Fthenakis, George C. In *Biology.* 2021 10(10) Language: English. DOI: 10.3390/biology10101016

Subjects: Staphylococcus aureus; ampicillin; **antibiotic resistance**; **biofilm**; bulk milk; clindamycin; cross-sectional studies; fosfomycin; lactation; management systems; milk quality; oxacillin; sheep; Greece

BU InfoLinks

EBSCO Connect | Privacy Policy | A/B Testing | Terms of Use

Copyright | Cookie Policy | Manage my Cookies

© 2024 EBSCO Industries, Inc. All rights reserved.
