# Supplementary material for: Antimicrobial Resistance and Residues from Biofilms in Poultry, Swine, and Cattle Farms: A Scoping Review
Source: Animals (Basel). 2025 Sep 22;15(18):2756. doi: 10.3390/ani15182756 (PMC12466552; doi:10.3390/ani15182756)
Supplement: Supplementary file 1 [file animals-15-02756-s001.zip › Supplementary material S3.pdf]

Web of Science Search Strategy (v0.1)

Database: All Databases

Entitlements:

- WOS: 1900 to 2024
- BCI: 1926 to 2024
- CCC: 1998 to 2024
- DRCI: 1900 to 2024
- DIIDW: 1966 to 2024
- GRANTS: 1953 to 2024
- KJD: 1980 to 2024
- MEDLINE: 1950 to 2024
- PPRN: 1991 to 2024
- PQDT: 1637 to 2024
- SCIELO: 2002 to 2024
- ZOOREC: 1864 to 2024

Searches:

1: TS=(pig* OR swine* OR weaner OR fattener OR sow OR piglet* OR boar OR boars OR chick* OR poultry* OR broiler* OR layer* OR turkey* OR duck* OR geese OR goose OR fowl* OR avian* OR bird* OR hen OR hens OR flock* OR cattle OR beef OR cow* OR calf OR calves OR heifer* OR bull* OR bovine OR dairy OR "food-producing animal*" OR "food producing animal*" OR "food animal*" OR "animal husbandry" OR "animal farming" OR "domestic animal*" OR livestock) and Preprint Citation Index (Exclude – Database) Date Run: Tue Apr 23 2024 08:13:07 GMT-0600 (heure normale du centre nord-américain) Results: 16306529

2: TS=(multidrug OR MDR OR "multi-drug" OR drug OR antibiotic* OR antimicrobial* OR "anti-microbial*" OR microbial* OR antibacterial* OR "anti-bacterial*" OR bacteria$) and Preprint Citation Index (Exclude – Database) Date Run: Tue Apr 23 2024 08:14:26 GMT-0600 (heure normale du centre nord-américain) Results: 18678889

3: TS=(resistance OR resistant OR sensibility OR susceptibility) and Preprint Citation Index (Exclude – Database) Date Run: Tue Apr 23 2024 08:14:46 GMT-0600 (heure normale du centre nord-américain) Results: 8227984

4: TS=("resistance gene*" OR ARG OR "AMR gene*" OR "resistance determinant*" OR "mobile

genetic element*" OR MGE) and Preprint Citation Index (Exclude – Database) Date Run: Tue Apr 23 2024 08:15:12 GMT-0600 (heure normale du centre nord-américain) Results: 225940

5: TS=(residue*) and Preprint Citation Index (Exclude – Database) Date Run: Tue Apr 23 2024 08:15:29 GMT-0600 (heure normale du centre nord-américain) Results: 1834588

6: TS=(biofilm*) and Preprint Citation Index (Exclude – Database) Date Run: Tue Apr 23 2024 08:15:51 GMT-0600 (heure normale du centre nord-américain) Results: 196526

7: TS=(farm* or “farm-level”) and Preprint Citation Index (Exclude – Database) Date Run: Tue Apr 23 2024 08:16:13 GMT-0600 (heure normale du centre nord-américain) Results: 1051946

8: #2 AND #3 and Preprint Citation Index (Exclude – Database) Date Run: Tue Apr 23 2024 08:17:19 GMT-0600 (heure normale du centre nord-américain) Results: 2277350

9: #5 AND #2 and Preprint Citation Index (Exclude – Database) Date Run: Tue Apr 23 2024 08:17:36 GMT-0600 (heure normale du centre nord-américain) Results: 571381

10: #4 OR #8 OR #9 and Preprint Citation Index (Exclude – Database) Date Run: Tue Apr 23 2024 08:19:13 GMT-0600 (heure normale du centre nord-américain) Results: 2898421

11: #4 OR #8 OR #9 and Preprint Citation Index (Exclude – Database) Date Run: Tue Apr 23 2024 08:19:17 GMT-0600 (heure normale du centre nord-américain) Results: 2898421

12: #11 AND #1 AND #6 AND #7 and Preprint Citation Index (Exclude – Database) Date Run: Tue Apr 23 2024 08:19:48 GMT-0600 (heure normale du centre nord-américain) Results: 618

13: #11 AND #1 AND #6 AND #7 and Preprint Citation Index (Exclude – Database) Date Run: Tue Apr 23 2024 08:19:54 GMT-0600 (heure normale du centre nord-américain) Results: 618
